# Protective effect of the oral administration of cystine and theanine on oxaliplatin-induced peripheral neuropathy: a pilot randomized trial

**DOI:** 10.1007/s10147-020-01728-4

**Published:** 2020-06-27

**Authors:** Minoru Kobayashi, Ryuichiro Sato, Toshihiro Komura, Hidetaka Ichikawa, Tomoaki Hirashima, Satoshi Otake, Naoya Akazawa, Takashi Yazawa, Tomoya Abe, Takaho Okada, Tetsuya Kakita, Masaya Oikawa, Takashi Tsuchiya

**Affiliations:** 1 Department of Surgery, Sendai City Medical Center, 5-22-1 Tsurugaya, Miyagino-ku, Sendai, Miyagi 983-0824 Japan; 2 Department of Surgery, Tohoku University Graduate School of Medicine, Sendai, Miyagi Japan; 3 Department of Surgery, Omagari Kousei Medical Center, Akita, Akita Japan

**Keywords:** Colorectal cancer, Neuropathy, Oxaliplatin, CIPN, Amino acid, Cystine, Theanine

## Abstract

**Background:**

Oxaliplatin, one of the key cytotoxic drugs for colorectal cancer, frequently causes peripheral neuropathy which leads to dose modification and decreased patients’ quality of life. However, prophylactic or therapeutic measures have not yet been established. Orally administered amino acids, cystine and theanine, promoted the synthesis of glutathione which was one of the potential candidates for preventing the neuropathy. The aim of this study was to determine whether daily oral administration of cystine and theanine attenuated oxaliplatin-induced peripheral neuropathy (OXLIPN).

**Methods:**

Twenty-eight colorectal cancer patients who received infusional 5-fluorouracil, leucovorin, and oxaliplatin (mFOLFOX6) therapy were randomly and evenly assigned to the cystine and theanine group and the control group. OXLIPN was assessed up to the sixth course using original 7-item questionnaire as well as Common Terminology Criteria for Adverse Events (CTCAE) grading scale.

**Results:**

Neuropathy scores according to our original questionnaire were significantly smaller in the cystine and theanine group at the fourth (*p* = 0.026), fifth (*p* = 0.029), and sixth course (*p* = 0.038). Furthermore, significant differences were also observed in CTCAE neuropathy grades at the fourth (*p* = 0.037) and the sixth course (*p* = 0.017). There was one patient in each group who required dose reduction due to OXLIPN. Except for neurotoxicity, no significant differences were noted in the incidence of adverse events, and the total amount of administered oxaliplatin.

**Conclusion:**

The results demonstrated the daily oral administration of cystine and theanine attenuated OXLIPN.

**Electronic supplementary material:**

The online version of this article (10.1007/s10147-020-01728-4) contains supplementary material, which is available to authorized users.

## Introduction

Colorectal cancer (CRC) is one of the most common cancers in the world. In 2018, an estimated 1,800,000 cases were newly diagnosed and nearly 860,000 patients died of the disease [1]. Surgical resection is the only curative treatment of localized CRC, and advanced disease is often treated with a combination of chemotherapy, radiation therapy, and surgery. The chemotherapeutic efficacy in CRC has improved since the introduction of combination regimens including 5-fluorouracil (5-FU) and either oxaliplatin (L-OHP) or irinotecan [2].

L-OHP, synthesized in 1976, is a platinum-based cytotoxic drug [3]. It is used to treat several cancers including CRC, and combination chemotherapies with L-OHP have demonstrated significant activity against advanced CRC. On the other hand, oxaliplatin-induced peripheral neuropathy (OXLIPN) is a common side effect which results in dose modification or discontinuation of the chemotherapy. It also impairs patients’ quality of life (QOL). OXLIPN can be classified into two different types: early-onset and late-onset neuropathy [4]. The former is characterized by acute and transient cold hyperesthesia, which occurs immediately after L-OHP infusion. The latter is a dose-dependent chronic cumulative peripheral neuropathy without motor involvement. Pachman et al. reported that 308 (89%) of 346 patients who received FOLFOX (5-FU, leucovorin, and L-OHP) therapy experienced acute neuropathy [5]. In the multicenter phase III study (MOSAIC study), grade two or higher peripheral neuropathy was observed in 229 (20.9%) of 1092 patients 1 month after 12 cycles of FOLFOX therapy [6]. Gebremedhn et al. reported that about 10–20% of patients who were treated with L-OHP-based chemotherapy for advanced CRC required dose reduction because of OXLIPN in each cycle [7]. In order to improve patients’ QOL and administer the L-OHP-containing chemotherapies as scheduled, it is important to prevent and mitigate OXLIPN. Over the past few decades, many studies have investigated the prevention and treatment of chemotherapy-induced peripheral neuropathy (CIPN), but few agents have been demonstrated to be effective. The American Society of Clinical Oncology (ASCO) guideline does not recommend any particular agents for the prevention of CIPN [8]. Developing novel preventive measures for OXLIPN is therefore very important.

Cystine and theanine is a supplement that contains 700 mg cystine and 280 mg theanine. After oral supplementation, cystine and theanine are changed into cysteine and glutamic acid, respectively, in the presence of glycine [9, 10]. Cysteine and glutamic acid are finally synthesized into glutathione (GSH) which is a strong antioxidant. GSH and its precursors, *N*-acetylcysteine and glutamine, were demonstrated to have protective effects against OXLIPN [11–14]. We previously reported that the oral administration of cystine and theanine reduced the incidence of S-1-associated adverse events (AEs) in patients who underwent curative surgery for either colon or gastric cancer [15]. However, the effect of cystine and theanine on OXLIPN has not been investigated. The aim of this study was to determine whether orally administrated cystine and theanine attenuated OXLIPN in CRC patients. AEs including OXLIPN were measured using Common Terminology Criteria for Adverse Events (CTCAE) grading scale which is an objective evaluation system [16]. Moreover, we created an original questionnaire which enabled precise evaluation of subjective symptoms of OXLIPN.

## Patients and methods

### Study design and patients

This study was a prospective randomized trial in CRC patients who received mFOLFOX6 therapy between 2015 and 2016 at the Sendai City Medical Center (UMIN 000020456). The study was approved by the institutional review board (approval number: 2015-0029), and all the participants provided written informed consent. The study was performed in accordance with the Declaration of Helsinki. Eligible patients were from 20 to 80 years old and in performance status 0 or 1 [17]. The participants were treated as first-line chemotherapy for unresectable disease or as post-operative adjuvant chemotherapy. Participants were excluded if they had a documented medical history of active infection, poorly controlled hypertension or diabetes, severe cardiac or pulmonary disease, clinically important psychopathic disorder or central nervous system damage, the continuous systemic administration of steroids, pregnancy, breastfeeding, impossibility of oral ingestion, and markedly abnormal renal or liver function tests. Patients with a history of prior chemotherapy were also excluded. Patients met the following criteria regarding their pre-registration laboratory tests: white blood cell count > 3000/mm^3^, neutrophil count > 1500/mm^3^, platelet count > 75,000/mm^3^, and hemoglobin level > 9.0 g/dl. Concomitant use of analgesics or moisturizing agents was allowed, but taking other amino acid supplements during this study was prohibited.

### Randomization and treatment

Since the study was exploratory, the sample size was determined as the highest-possible number of participants during the research period. The subjects were divided into cystine and theanine group (C/T group) and control group, and allocation was generated using the envelope method. In total, 28 patients were included in the study, with 14 in each group. The patients in the C/T group were orally administered cystine (700 mg) and theanine (280 mg) (Ajinomoto, Tokyo, Japan) once a day during the study. The patients in the control group received mFOLFOX6 therapy without cystine and theanine. All participants received mFOLFOX6 therapy, once every 2 weeks: L-OHP 85 mg/m^2^, levofolinate calcium 200 mg/m^2^, bolus 5-FU 400 mg/m^2^, all on day 1; infusion 5-FU 2400 mg/m^2^ on day 1–3 (Supplementary Fig. 1). A molecular target drug, bevacizumab, was administered on day 1 when indicated. The maximum number of courses was six in this study.

### Assessments

To assess the degree of peripheral neuropathy, we created a 7-item questionnaire based on the Functional Assessment of Cancer Therapy/Gynecologic Oncology Group-Neurotoxicity (FACT/GOG-Ntx) Questionnaire [18] (Table 1). The score was ranging from 0 to 28, and the severity of neuropathy was assessed at the start of each cycle of chemotherapy. The peripheral neuropathy and other AEs were also evaluated according to CTCAE v4.0. Peripheral neuropathy grades in CTCAE include motor and sensory neuropathy grades, and the higher grade was chosen in the study. Dose reduction or the cessation of the chemotherapy was decided by the doctor-in-charge based on the degree of AEs. The study was stopped if the regimen was changed because of the severity of AEs or the progression of the disease. The primary endpoint was the suppressive effect of cystine and theanine on peripheral neuropathy during mFOLFOX6 therapy. Secondary endpoints were the frequency of other AEs of grade two or greater and the total amount of administered L-OHP.

**Table 1:** The questionnaire of peripheral neuropathy

	Not at all	A little bit	Some-what	Quite a bit	Very much
I have numbness or tingling in my hands	0	1	2	3	4
I have numbness or tingling in my feet	0	1	2	3	4
I have discomfort in my hands	0	1	2	3	4
I have discomfort in my feet	0	1	2	3	4
I have trouble buttoning buttons	0	1	2	3	4
I have trouble feeling the shape of objects in my hand	0	1	2	3	4
I have trouble walking	0	1	2	3	4

### Statistical analysis

The Mann–Whitney *U* test was used to compare the values, and the χ^2^ test was used to compare patients’ backgrounds between the two groups. Reliability of the scale was measured by the Cronbach’s *α* coefficient. Spearman’s rank correlation coefficient was used to evaluate correlations. All data were presented as a number of the cases or mean ± standard error of the mean (SEM). A *p* value below 0.05 was considered statistically significant. Statistical analysis was performed with BellCurve for Excel ver.2.15 (Social Survey Research Information Co., Ltd., Japan).

## Results

### Patients

The characteristics of the patients were summarized in Table 2. The mean age of the patients in the C/T group and the control group was 58.1 and 67.5 years old, respectively, and patients in the C/T group were significantly younger (*p* = 0.012). The proportion of women was higher in the control group than in the C/T group (78.6% vs. 42.9%) and the difference was marginally significant (*p* = 0.053). Other factors including BMI, BSA, and stage were comparable between the groups. The completion rate for cystine and theanine in the treatment group was 100%. Eleven patients in the C/T group and nine patients in the control group received mFOLFOX6 as post-operative adjuvant chemotherapy while others were administered as first-line chemotherapy for the unresectable disease (*p* = 0.403). One patient in the C/T group and five patients in the control group were concomitantly administrated bevacizumab (*p* = 0.065).

**Table 2:** The characteristics of the patients

	C/T (*n* = 14)	Control (*n* = 14)	*p* value
Age (years)	58.1 ± 2.7	67.5 ± 1.7	0.012*
Sex, male:female (cases)	8:6	3:11	0.053
Body mass index (kg/m^2^)	21.5 ± 0.8	23.4 ± 0.8	0.108
Body surface area (m^2^)	1.63 ± 0.05	1.57 ± 0.04	0.462
Diabetes mellitus (cases)	1	2	0.541
Stage II:III:IV (cases)	0:9:5	3:7:4	0.186
Adjuvant chemotherapy (cases)	11	9	0.403

Stage was evaluated by the seventh edition of the TNM Classification of Malignant tumours

*C/T* cystine and theanine

*p < 0.05

### Original 7-item questionnaire

The collection rate of the original 7-item questionnaire in the study was 96.7%. The Cronbach’s *α* coefficient of internal consistency for the questionnaire was 0.78. The spearman rank-correlation coefficient between our original scores and CTCAE neurosensory toxicity grades was 0.60 (*p* < 0.001).

### Adverse events

**Fig. 1:**
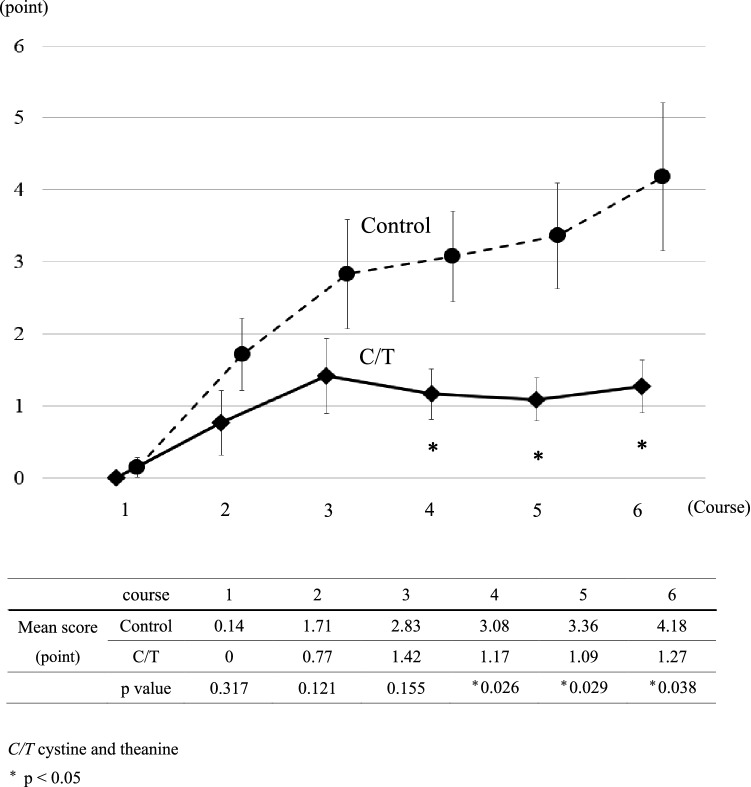
The scores of the peripheral neuropathy. The solid and dotted line show the mean score of peripheral neuropathy according to our original questionnaire at the start of each cycle in the C/T group and the control group, respectively. The error bars represent standard error of the mean

**Fig. 2:**
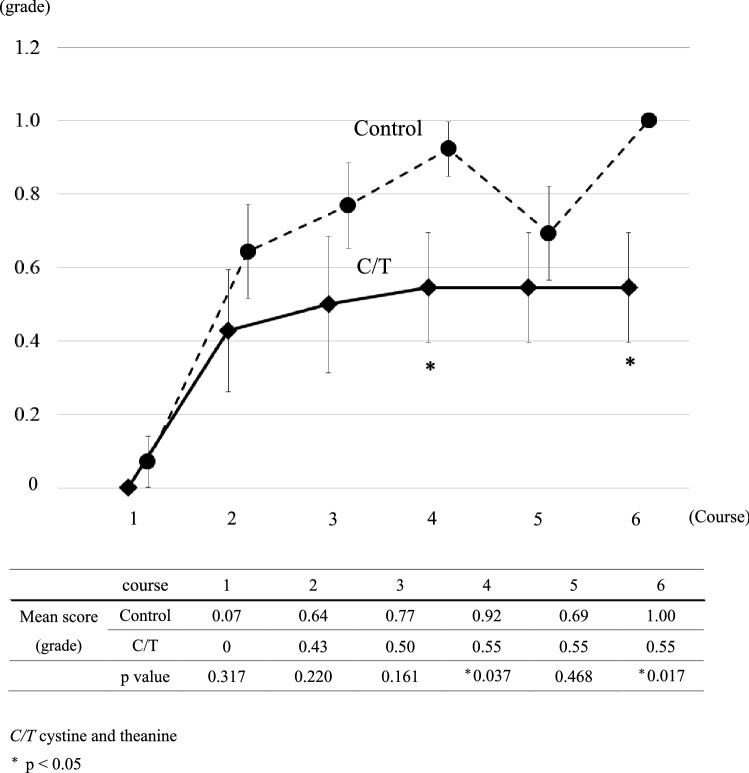
CTCAE neuropathy grades. The solid and dotted line show the mean grade of peripheral neuropathy according to the CTCAE at the start of each cycle in the C/T group and the control group, respectively. The error bars represent standard error of the mean

Neuropathy scores according to our original questionnaire were significantly smaller in the C/T group than in the control group at the fourth (1.17 and 3.08, respectively, *p* = 0.026), fifth (1.09 and 3.36, respectively, *p* = 0.029), and sixth course (1.27 and 4.18, respectively, *p* = 0.038) (Fig. 1). Furthermore, significant differences were also observed in CTCAE neuropathy grades at the fourth (0.55 and 0.92, respectively, *p* = 0.037) and the sixth course (0.55 and 1.00, respectively, *p* = 0.017) (Fig. 2). Except for neurotoxicity, AEs greater than grade two were present in ten patients in each group, and the incidences of each AE were not different between the groups (Table 3). Grade three AEs were reported in three patients in the C/T group and in four patients in the control group (*p* = 0.66), and there were no AEs greater than grade four in each group. There were no AEs attributable to cystine and theanine in this study.

**Table 3:** The incidence of adverse events

	C/T (*n* = 14)	Control (*n* = 14)	*p* value
AEs grade > 2	10	10	1.00
Leukopenia	7	6	0.71
Neutropenia	8	8	1.00
Thrombocytopenia	0	2	0.14
Appetite loss	2	1	0.54
Nausea	1	1	1.00
Diarrhea	0	1	0.30
Constipation	1	0	0.30
Non-cardiac chest pain	0	1	0.30
AEs grade > 3	3	4	0.66
Leukopenia	1	3	0.28
Neutropenia	3	4	0.66

*C/T* cystine and theanine, *AEs* adverse events

### Chemotherapy dose and courses

The cumulative dose of L-OHP was 671 ± 52 mg in the C/T group and 714 ± 23 mg in the control group, which was not statistically different (*p* = 0.85). The L-OHP dose was reduced in six patients in the C/T group and in seven patients in the control group. Of these, there was one patient in each group who required reduction of L-OHP dose due to peripheral neuropathy. The ratio of the cumulative dose to the predetermined dose in the C/T group and the control group was 90.5% and 94.7%, respectively (*p* = 0.98). The number of patients who completed six courses without suspension was 11 in the C/T group and 12 in the control group (*p* = 0.62). In each group, one patient did not complete six courses of mFOLFOX6, because of multiple AEs in the C/T group and continuous chest pain in the control group.

### Other supportive agents

Patients who received pregabalin for neuropathic pain were five in the C/T group and six in the control group (*p* = 0.70). Three patients in the C/T group and seven in the control group prescribed moisturizing agents for the prevention of dry skin (*p* = 0.11). Duloxetine, which was moderately recommended for the treatment of CIPN by ASCO guidelines [8], had been administered to one patient in the control group for treatment of depression before the study began. There was no new prescription of duloxetine in both groups during the study.

## Discussion

In the present study, we investigated the protective effect of orally administered cystine and theanine on OXLIPN in CRC patients who received mFOLFOX6 chemotherapy. Neuropathy scores according to our original questionnaire and the CTCAE neuropathy grades were significantly smaller in the C/T group. Cystine and theanine promoted the synthesis of GSH which was one of the potential candidates for the prevention of CIPN. Cascinu et al. demonstrated that GSH had protective effects against OXLIPN in CRC patients [11]. Lin et al. showed that the oral administration of *N*-acetylcysteine, which is a precursor of GSH, attenuated OXLIPN [13], and Wang et al. revealed that the oral intake of glutamine, which is another precursor, was effective for preventing OXLIPN [14]. Although the efficacy of GSH has been somewhat inconclusive, the result of the present study indicated orally administrated cystine and theanine suppressed OXLIPN presumably through GSH in much the same way as *N*-acetylcysteine and glutamine.

GSH has antioxidant properties, but previous study demonstrated GSH did not attenuate L-OHP activity. In a randomized study of 52 CRC patients who were treated with L-OHP-based regimen, GSH significantly reduced OXLIPN without affecting tumor response rate, progression-free survival, and overall survival [11]. In a mouse xenograft model, cystine and theanine did not reduce cisplatin anti-tumor activity nor stimulate tumor growth (personal communication). In the previous studies, GSH was generally given intravenously only once before the administration of anticancer drugs [11, 12, 19–21]. However, Hong et al. demonstrated the elimination half-life of GSH in serum following intravenous injection was about 11 min [22], which indicated that the GSH concentration 60 min after the administration was equal to its normal level. In contrast, the elimination half-life of L-OHP exhibited three phases: the alpha half-life was about 23 min, the beta half-life was about 12 h, and the gamma half-life was 152 h when L-OHP was administered at 90 mg/m^2^ [23]. Therefore, the concentration of L-OHP might remain at a relatively high level when that of GSH decreased. On the other hand, it is presumed that the daily oral administration of GSH precursors could keep the blood concentration of GSH at a certain level, which might result in a stronger protective effect on OXLIPN compared to single intravenous administration of GSH. The “cystine and theanine” is a promising candidate for OXLPN prevention with potential advantages over GSH since it is an inexpensive, commercially available oral supplement.

In order to evaluate neurotoxicity, the CTCAE or World Health Organization (WHO) guidelines were used in the previous studies which demonstrated the effect of GSH or its precursors on CIPN [11–14, 20, 21, 24, 25]. These grading systems are five-grade evaluation systems, which might not appropriate to evaluate subtle change of peripheral neuropathy [26, 27], and they are healthcare provider-based assessment tools that can be interpreted differently between observers [28]. We created an original questionnaire based on FACT/GOG-Ntx which was recommended by a systematic review on the assessment tools of CIPN [29]. The FACT/GOG-NTx subscale was comprised of 11 items, and it was validated in patients with ovarian cancer who were treated with taxane- and platinum-based regimen [18]. Kopec et al. assessed neurologic symptoms of colon cancer patients receiving L-OHP-containing regimen with modified FACT/GOG-Ntx, and they also validated the scale by demonstrating a significant correlation with the NCI-Sanofi criteria which is a modified CTCAE [30]. We selected seven items that had demonstrated strong correlations in their study and developed our original scale. The spearman rank-correlation coefficient between our original scores and CTCAE grades was 0.60 (*p* < 0.001). Hausheer et al. claimed that CIPN assessment tool should be easy to use, and that the collection rate should be greater than 80% [31]. The collection rate of the questionnaire in the study was more than 90%, which could be partly attributed to the simplified scoring system. Neuropathy scores according to our original questionnaire were significantly different between the two groups at the fifth course, whereas CTCAE grade failed to demonstrate a significant difference. These findings indicated that our scoring system was easy to use and was more sensitive than CTCAE in detecting mild changes in neuropathy.

The result of the present study showed the oral administration of cystine and theanine significantly attenuated OXLIPN. We previously reported that cystine and theanine reduced the incidence of S-1-associated diarrhea [15]. Meanwhile, a statistically significant difference in the incidence of each AE was not found except for peripheral neuropathy in this study, which was partly because the incidence of diarrhea was relatively low. Recently, combination chemotherapy with S-1 and L-OHP (SOX therapy) has been used for the treatment of advanced gastric and CRC patients [32, 33], in which diarrhea and peripheral neuropathy were frequently observed. In this respect, our results suggested that cystine and theanine could be potential adjunct agents for SOX therapy.

This study has several limitations. The sample size was small and the study was neither double-blinded nor placebo-controlled. Moreover, significant difference was observed in patients age, and female predominance in the control group was marginally significant, although these differences were not correlated with peripheral neuropathy scores (data not shown). The study duration of six courses and L-OHP cumulative dose of around 700 mg might not be enough to fully assess the potential impacts of cystine and theanine. It is necessary to plan a large, well-designed additional study. However, this study was of great importance since this was the first to demonstrate that the daily oral administration of cystine and theanine attenuated OXLIPN. Further research is needed to reduce peripheral neuropathy and to improve the QOL of patients who receive L-OHP based chemotherapy.

## Electronic supplementary material

Below is the link to the electronic supplementary material.

Supplementary file1 (PDF 55 kb)
