# Supplementary material for: Protective effect of the oral administration of cystine and theanine on oxaliplatin-induced peripheral neuropathy: a pilot randomized trial
Source: Int J Clin Oncol. 2020 Jun 27;25(10):1814–21. doi: 10.1007/s10147-020-01728-4 (PMC7498479; doi:10.1007/s10147-020-01728-4)
Supplement: Supplementary file 1 — Supplementary file1 (PDF 55 kb) [file 10147_2020_1728_MOESM1_ESM.pdf]

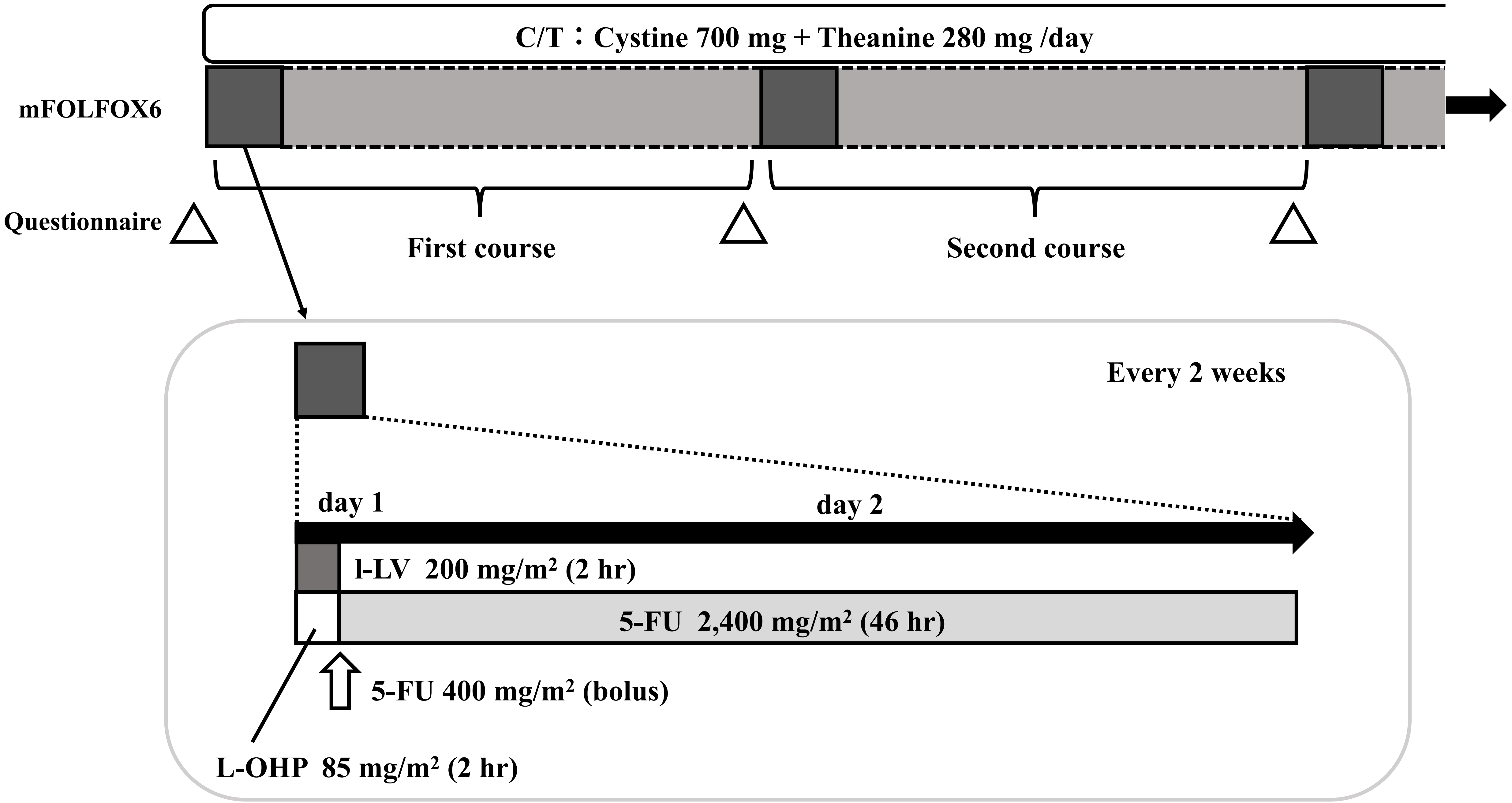

Supplementary Figure 1 The study schedule of mFOLFOX6 and the supplementation of cystine and theanine
